# Wearable laser Doppler flowmetry for non-invasive assessment of diabetic foot microcirculation: methodological considerations and clinical implications

**DOI:** 10.1117/1.JBO.29.6.065001

**Published:** 2024-05-11

**Authors:** Xing-Xi Hu, Xiao-Man Xing, Zhen-Ming Zhang, Chao Zhang, Li Chen, Jia-Zhang Huang, Xu Wang, Xin Ma, Xiang Geng

**Affiliations:** a Huashan Hospital, Fudan University, Department of Orthopedic Surgery, Shanghai, China; b The Affiliated Hospital of Yunnan University (The Second People’s Hospital of Yunnan Province, The Eye Hospital of Yunnan Province), Department of Orthopedics and Trauma, Kunming, China; c University of Science and Technology of China, School of Biomedical Engineering (Suzhou), Division of Life Sciences and Medicine, Suzhou, China; d Chinese Academy of Sciences, Suzhou Institute of Biomedical Engineering and Technology, Suzhou, China; e National Center for Orthopaedics, Shanghai Sixth People’s Hospital Affiliated to Shanghai Jiao Tong University School of Medicine, Shanghai, China

**Keywords:** laser Doppler flowmetry, type 2 diabetes mellitus, wavelet analysis, sample entropy, foot microcirculation

## Abstract

**Significance:**

Type 2 diabetes mellitus (T2DM) is a global health concern with significant implications for vascular health. The current evaluation methods cannot achieve effective, portable, and quantitative evaluation of foot microcirculation.

**Aim:**

We aim to use a wearable device laser Doppler flowmetry (LDF) to evaluate the foot microcirculation of T2DM patients at rest.

**Approach:**

Eleven T2DM patients and twelve healthy subjects participated in this study. The wearable LDF was used to measure the blood flows (BFs) for regions of the first metatarsal head (M1), fifth metatarsal head (M5), heel, and dorsal foot. Typical wavelet analysis was used to decompose the five individual control mechanisms: endothelial, neurogenic, myogenic, respiratory, and heart components. The mean BF and sample entropy (SE) were calculated, and the differences between diabetic patients and healthy adults and among the four regions were compared.

**Results:**

Diabetic patients showed significantly reduced mean BF in the neurogenic (p=0.044) and heart (p=0.001) components at the M1 and M5 regions (p=0.025) compared with healthy adults. Diabetic patients had significantly lower SE in the neurogenic (p=0.049) and myogenic (p=0.032) components at the M1 region, as well as in the endothelial (p<0.001) component at the M5 region and in the myogenic component at the dorsal foot (p=0.007), compared with healthy adults. The SE in the myogenic component at the dorsal foot was lower than at the M5 region (p=0.050) and heel area (p=0.041). Similarly, the SE in the heart component at the dorsal foot was lower than at the M5 region (p=0.017) and heel area (p=0.028) in diabetic patients.

**Conclusions:**

This study indicated the potential of using the novel wearable LDF device for tracking vascular complications and implementing targeted interventions in T2DM patients.

## 1 Introduction

Diabetes mellitus (DM) is a significant global health issue, impacting millions worldwide. In 2021, the International Diabetes Federation estimated that the global prevalence of diabetes among individuals aged 20 to 79 years stood at 10.5%, equating to 536.6 million affected individuals. Projections indicate that this figure will further escalate to 12.2% (783.2 million people) by 2045.^1^ China has the highest number of DM patients, comprising approximately one-third of the global total.^2^

One of the most common and serious complications of DM is diabetic foot (DF), which occurs in ∼15% to 25% of DM patients.^3^ DF is characterized by foot ulceration, infection, and/or gangrene, which can ultimately lead to amputation or even mortality.^4^ The development of DF involves multiple factors, including peripheral neuropathy, hyperglycemia, microvascular damage, impaired angiogenesis, altered biomechanics of plantar soft tissue, and abnormal gait resulting in increased plantar pressure and shear stress.^4^ Among these factors, microvascular dysfunction leading to plantar tissue ischemia is widely acknowledged as a crucial contributor to the formation of DF ulcers (DFUs).^5^

The microcirculation can be assessed by various optical diagnostic methods, among which the most commonly used are laser speckle, video capillaroscopy, optical coherence tomography, and laser Doppler flowmetry (LDF).^6^ Among them, LDF can evaluate the blood flow (BF) of microvessels in the human body. Its basis is to use a laser to perform optical non-invasive radiation sensing on tissues and further analyze the scattering partially reflected by moving red blood cells.^7^ The signal measured by LDF based on wavelet analysis has specific rhythmic components (0.01 to 2 Hz), revealing five characteristic frequencies pertaining to endothelial-regulated BF dynamics, neurogenic control, myogenic contractions, respiratory rate, and cardiac activity. This method can facilitate our understanding of the potential mechanisms of impaired microvascular reactivity and can be used to detect diabetic patients with foot ulcer risk early and intervene actively, as much as possible to reduce the occurrence of foot ulcers. Wavelet analysis has good time resolution at high frequencies and good frequency resolution at low frequencies. This multi-resolution time-frequency analysis has been proven to be a good method for analyzing non-stationary biological signals in early studies, such as electromyogram^8^ and skin BF^9^ signals. Wavelet analysis of BF oscillations has great potential to facilitate understanding of the control mechanisms of skin BF. Jan et al.^10^ included 18 type 2 diabetic patients (DM2) with peripheral neuropathy and 8 healthy controls. Skin BF at M1 was measured by LDF under 300 mmHg mechanical stress and 42°C rapid thermal stress. Wavelet analysis was used to evaluate metabolic, neurogenic, and myogenic control. The results suggested that diabetes causes damage to metabolic, neurogenic, and myogenic control, leading to microvascular dysfunction. Another study by Zherebtsov et al.^11^ also demonstrated that the area under the continuous wavelet spectrum in the major frequency ranges held significant diagnostic value for detecting microvascular complications in DM patients. The team used autocorrelation analysis to study BF fluctuations in the microcirculation and successfully distinguished between healthy young and elderly subjects, as well as diabetic patients. Local mechanical and thermal stress can be used to evaluate microvascular reactivity and the risk of DFUs. Mizeva et al.^12^ studied 40 healthy subjects, 17 type 1 diabetic patients (DM1) and 23 DM2. Skin BF was collected by LDF. One foot was cooled to 25°C for 4 min, and local thermal tests were performed at 35°C and 42°C for 4 and 10 min, respectively. The results suggested that local temperature tests showed impaired vascular dilation function in response to local heating in diabetic patients. A trend of impaired low-frequency BF pulsations related to endothelial and neurogenic activity was observed in both groups of diabetic patients. Saha et al.^13^ recorded the blood circulation of healthy young non-smokers and smokers using a wearable LDF device. The results suggested that the blood perfusion level of the non-smoking group was higher than that of the smoking group, which can be used to evaluate the sensitivity of wearable LDF sensors in determining the effect of nicotine on smokers and non-smokers and the blood microcirculation of smokers with different diseases.

Currently, studies investigating diabetic microcirculation using LDF are primarily conducted in laboratory settings, which are limited by experimental conditions and environmental constraints. Although wearable LDF devices have been available for some time, there is a lack of research utilizing them to compare plantar microcirculation between healthy individuals and those with type 2 diabetes. While wearable LDF is convenient, it does have limitations:

1. Reduced accuracy and precision: Wearable LDF devices may exhibit lower accuracy and precision compared with traditional laboratory-based LDF systems. Factors such as motion artifacts, ambient light interference, and variations in device placement or contact with the skin can affect the reliability of measurements.
2. Limited depth penetration: Wearable LDF devices typically have shallow depth penetration. According to Zharkikh et al.,^14^ who investigated the depth sensitivity of LDF, the method typically achieves a penetration depth of more than 2 mm, with a source-detector separation of more than 1 mm. This limitation may restrict their ability to measure microcirculation and hinder the assessment of perfusion in deeper tissues, which can be relevant in certain clinical scenarios.
3. Potential for measurement variability: The nature of wearable LDF devices, influenced by external factors such as device placement and contact pressure, introduces the risk of measurement variability among individuals or even within the same individual over time. This variability can impact the reliability and comparability of results.

It is important to consider these disadvantages when using wearable LDF devices and interpreting the results. Appropriate validation and standardization measures should be implemented to overcome these limitations.

Therefore, the objectives of this study are as follows:

1. Evaluate the early monitoring potential of wearable LDF for assessing diabetic microcirculation, employing wavelet analysis to uncover mechanisms underlying impaired reactivity and aid in the development of strategies for preventing DFU.
2. Contrast foot microcirculation between healthy individuals and diabetics using wearable LDF, deepening our understanding of the microvascular effects associated with diabetes.
3. Investigate regional differences in microcirculation within and between healthy and diabetic feet, providing a comprehensive view of diabetes-induced alterations in microvasculature.

## 2 Materials and Methods

### 2.1 Study Subjects

This study will include the following two groups of subjects as study objects: (1) healthy middle-aged and elderly people (healthy older group): aged between 50 and 70 years, with no history of diabetes; (2) diabetic subjects (diabetes group): age-matched (between 50 and 70 years) type II diabetic patients, with clear diagnosis [according to the diagnostic criteria of “China Type 2 Diabetes Prevention and Treatment Guidelines (2020 Edition)”^15^]. The inclusion criteria include: (1) adult aged 50 to 70 years; (2) able to complete normal gait cycle independently and cooperate with physical examination and related tests; (3) no lower limb-related diseases that may affect plantar mechanical distribution other than diabetes; and (4) diabetic patients without foot ulcers or ulcer history. The exclusion criteria include: (1) the presence of lower limb-related diseases, such as DF, foot and ankle deformity, heel pain syndrome, stroke, and knee/arthritis, which may affect gait; (2) the presence of foot ulcers. The subjects were enrolled strictly in accordance with the Helsinki Declaration and approved by the Ethics Committee of Huashan Hospital Affiliated to Fudan University. All subjects were enrolled after paper informed consent.

The study included a total of 11 diabetic patients (four males and seven females) and 12 healthy adults (six males and six females). The diabetic patients had an average duration of diabetes of 4.9±3.6 years, fasting blood glucose levels of 7.91±0.95  mmol/L, postprandial 2-h blood glucose levels of 12.70±4.92  mmol/L, and glycosylated hemoglobin levels of 7.25±1.25%.

There were no significant differences in mean age (63.3±7.0 versus 59.6±5.6 years), body mass index (BMI) (BMI; 23.2±3.9 versus 25.1±4.9), and ankle-brachial index (ABI; 1.16±0.11 versus 1.17±0.08) between the healthy adults and diabetic patients.

The average foot skin temperature of diabetic patients (28.17±1.24°C) was significantly lower than that of healthy adults (29.69±1.05°C; p=0.004). In diabetic patients, the positive numbers of monofilament touch, tuning fork vibration sense, and temperature sense tests were 4/11 (36.4%), 5/11 (45.5%), and 8/11 (72.7%), respectively.

For further details, please refer to Table 1, which summarizes the characteristics of the study participants.

**Table 1:** Baseline characteristics of the participants.

Baseline characteristics	Healthy adults (n=12)	Diabetic patients (n=11)	p-Value
Gender			0.434
Male	6	4	
Female	6	7	
Age	63.3 ± 7.0	59.6 ± 5.6	0.320
BMI	23.2 ± 3.9	25.1 ± 4.9	0.424
Smoking history			
Yes	4	2	0.408
No	8	9	
Drinking history			0.538
Yes	2	3	
No	10	8	
Duration of diabetes (years)	—	4.9 ± 3.6	—
Average foot skin temperature (°C)	29.69 ± 1.05	28.17 ± 1.24	0.004**
Dorsalis pedis artery pulse			0.231
Normal/good	1	3	
Diminished/palpable	11	8	
Posterior tibial artery pulse			0.552
Normal/good	4	5	
Diminished/palpable	8	6	
Fasting blood glucose (mmol/L)	—	7.91 ± 0.95	—
Postprandial blood glucose at 2 h (mmol/L)	—	12.70 ± 4.92	—
Glycated hemoglobin (%)	—	7.25 ± 1.25	—
Ankle-brachial index (ABI)	1.16 ± 0.11	1.17 ± 0.08	0.854
Monofilament test			—
Normal	—	7	63.6%
Abnormal	—	4	36.4%
Tuning fork vibration test			—
Normal	—	6	54.5%
Abnormal	—	5	45.5%
Thermocool test			—
Normal	—	3	27.3%
Abnormal	—	8	72.7%

**p<0.010

### 2.2 Experimental Steps

**Fig. 1:**
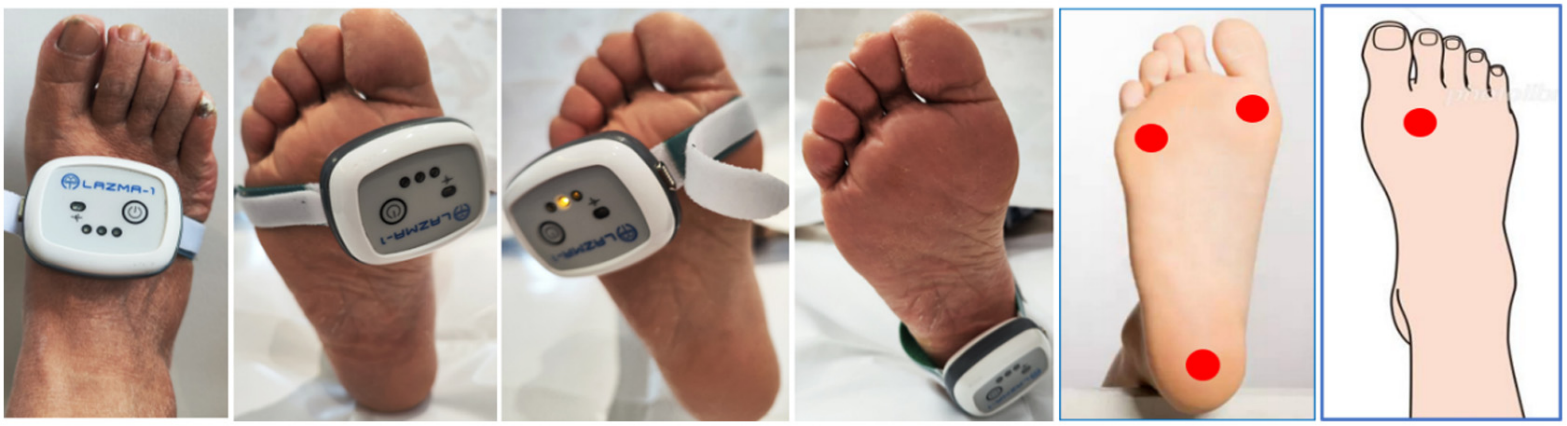
Test equipment and parts below M1, below M5, heel, dorsum of the foot, and between the first and second metatarsals.

1. Basic information and physical examination: Subjects who met the inclusion and exclusion criteria underwent a rigorous screening process, during which their baseline information and laboratory tests were collected, as shown in Table S1 in the Supplementary Material. Peripheral nerve damage was assessed using the following methods: Skin touch test: A 10g-Semmes-Weinstein nylon monofilament from Wuhu Baiyao Science and Education Instrument Co., Ltd., was used; Skin vibration sense: The Rydel-Seiffer semi-quantitative vibration tuning fork from Wuhu Baiyao Science and Education Instrument Co., Ltd. was employed; Temperature sense: A temperature sensation tester from Wuhu Baiyao Science and Education Instrument Co., Ltd., was utilized.
2. Data collection: The BF perfusion in specific areas was quantified using the Wearable LDF (AMT LAZMA-1, Birmingham, UK), with the selection of these specific areas informed by Jan et al.^10^ and the practical guidelines on the prevention and management of diabetes-related foot disease (IWGDF 2023 update).^16^ The areas included M1, M5, heel, and dorsum of the foot between the first and second metatarsals. The device employed a VCSEL chip (850 nm, 1.4  mW/3.5  mA, Philips, The Netherlands) as a single-mode laser source, eliminating the need for fiber optics and allowing direct tissue illumination. The sampling frequency was set at 20 Hz. For a visual representation, please refer to Fig. 1. To ensure accurate measurements, participants were instructed to abstain from consuming caffeine or alcohol-containing beverages for at least 1 h and 12 h before the measurement time.
3. Experimental environment: Prior to the test, the subjects were provided with a 30-min rest period in a quiet and comfortable indoor environment maintained at a room temperature of 24±2°C. Subsequently, they lay down on the test bed to complete the test, with each part being continuously collected for a duration of 3 min.

### 2.3 Data Processing

In this study, the LDF BF signal was initially decomposed into separate components. Subsequently, the power of each band and its complexity measure were utilized as features to compare different measurement sites and distinguish between healthy and diabetic subjects. We strictly filter out abnormal data that can be caused by a variety of factors and only retain those data points that meet strict signal-to-noise ratio criteria and effectively represent microcycle characteristics.

#### 2.3.1 Wavelet analysis

The LDF BF signal was decomposed with continuous Morlet wavelets. The wavelet transform and analysis methodology were extensively described in previous studies.^17^ The characteristic components associated with individual control mechanisms are as follows: endothelial origin (0.008 to 0.02 Hz), neurogenic origin (0.02 to 0.05 Hz), myogenic origin (0.05 to 0.15 Hz), respiratory origin (0.15 to 0.4 Hz), and heart origin (0.4 to 2.0 Hz) (as depicted in Fig. S1 in the Supplementary Material). It is crucial to recognize that our study employed a 3-min recording interval; this duration may be insufficient for robust analysis of low-frequency components, particularly those related to endothelial and neurogenic control. Given the multiple measurement locations in our investigation and considering the potential for extended monitoring to cause discomfort and affect data quality in DF disease patients, we adopted an innovative preprocessing approach to mitigate these issues.

#### 2.3.2 Mean BF

The average value of local microcirculation BF within each component was calculated during the observation period. This calculation provides insights into the perfusion of microcirculation at the specific test site.

#### 2.3.3 Sample entropy

The concept of sample entropy (SE) was introduced by Richman and Moorman^18^ in 2000 as a measure of time series complexity. A smaller SE value indicates lower complexity and higher self-similarity in the time series. Consequently, SE can be employed to assess the stability of microcirculation waveforms. In our study, we applied the following procedure to the BF within the frequency range of 0.0095 to 2 Hz. Initially, we decomposed the data sequence using the ensemble empirical mode decomposition (EEMD) method,^19^ resulting in multiple oscillatory modes, each with a narrow bandwidth, as depicted in Fig. S2 in the Supplementary Material. Subsequently, we excluded oscillatory modes with frequencies below 0.0095 Hz or above 2 Hz to reconstruct the filtered data sequence. The SE algorithm^18^ was then applied to this filtered data. Liao and Jan^20^ demonstrated the stability of SE values when using parameters of m=2 to 5 and r=0.2× standard deviation (SD) of the data sequence for skin BF signals lasting 10 minutes or less. Hence, in our study, we identified the optimal embedded dimension (m=3) specifically for wearable LDF data and adopted r=0.2×SD as the parameter values for SE calculation.

### 2.4 Statistical Analysis

To analyze the continuous data of microcirculation BF and SE in different parts of the two groups of subjects, we conducted various statistical tests, including the following steps:

1. Normality analysis: The Shapiro–Wilk test (W test) was used to assess normal distribution. Descriptive statistics were reported as either “mean ± standard deviation” or “median and range” based on the distribution.
2. Group comparison: Normality and homogeneity of variance were tested using the W test and modified Bartlett’s test. If the data met both criteria, a two independent samples T-test was used. If they met normality but not homogeneity of variance, Welch’s T-test was used. For non-normal data, the two independent samples Wilcoxon rank sum test was applied.
3. Comparison among parts: Normality and homogeneity of variance were tested using the W test and modified Bartlett’s test. If both criteria were met, a one-way analysis of variance with the LSD method for post-hoc multiple comparison was used. Otherwise, the non-parametric Kruskal–Wallis test (H test) was applied, with Dunnett’s method for post-hoc multiple comparison. All statistical analyses were conducted using R language version 4.2.2, and statistical significance was set at p<0.05.

All statistical analyses were performed using R language version 4.2.2 (Foundation for Statistical Computing, Vienna, Austria). All statistical analyses were based on two-sided tests with p-values less than 0.05 as statistically significant differences.

## 3 Results

### 3.1 Comparison of Mean BF in Different Components Between Diabetic Patients and Healthy Adults

**Fig. 2:**
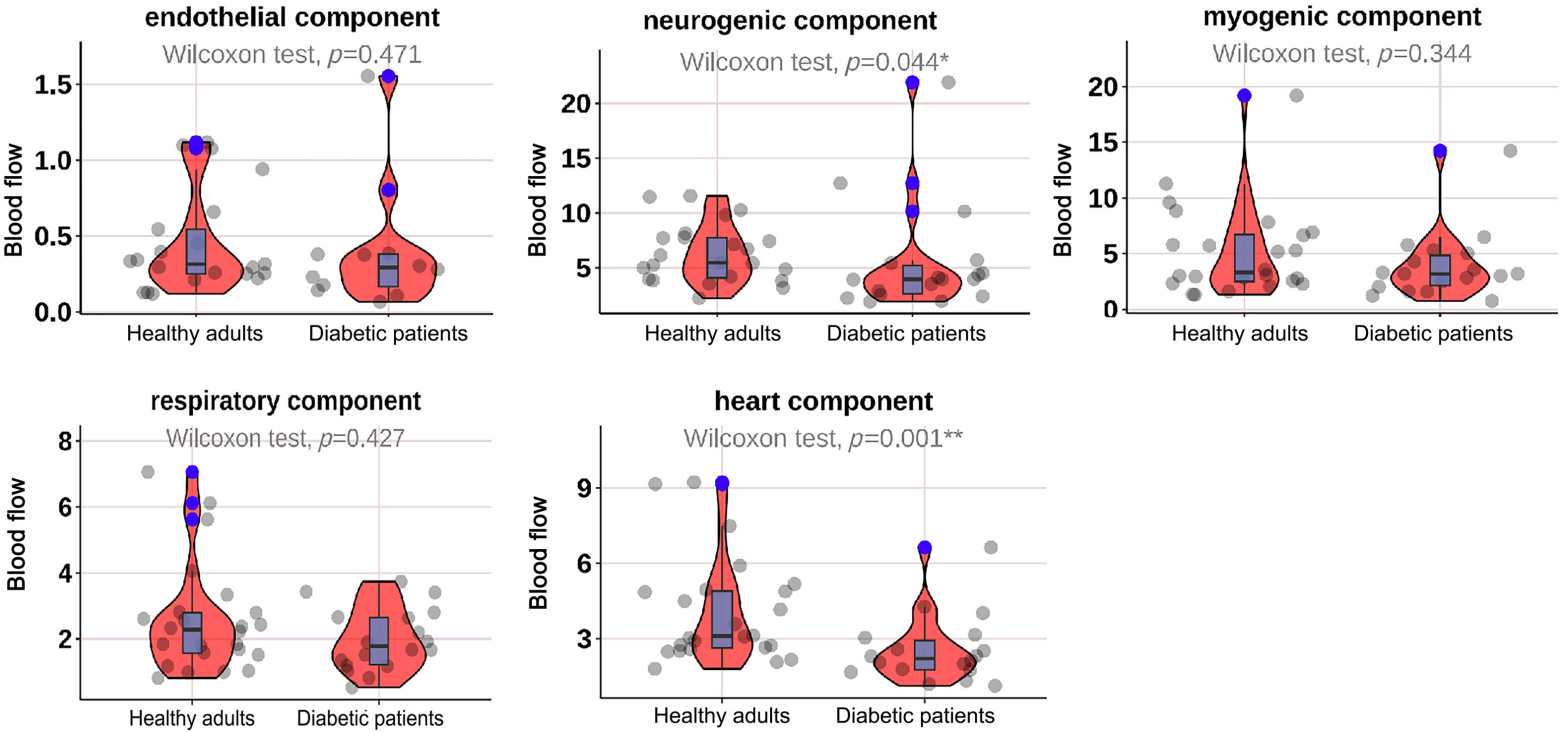
Violin plot comparing the time-integrated BF values of different wavelet components in the M1 region between diabetic patients and healthy adults. The neurogenic (p=0.044*) and heart (p=0.001**) components in diabetic patients were significantly lower than those in healthy adults, whereas there was no significant difference in the endothelial, myogenic, and respiratory components. The box plot represents the median and interquartile range, the gray scatter represents the BF of each subject, the blue scatter represents outliers, and the red kernel density plot represents the distribution of data density.

**Fig. 3:**
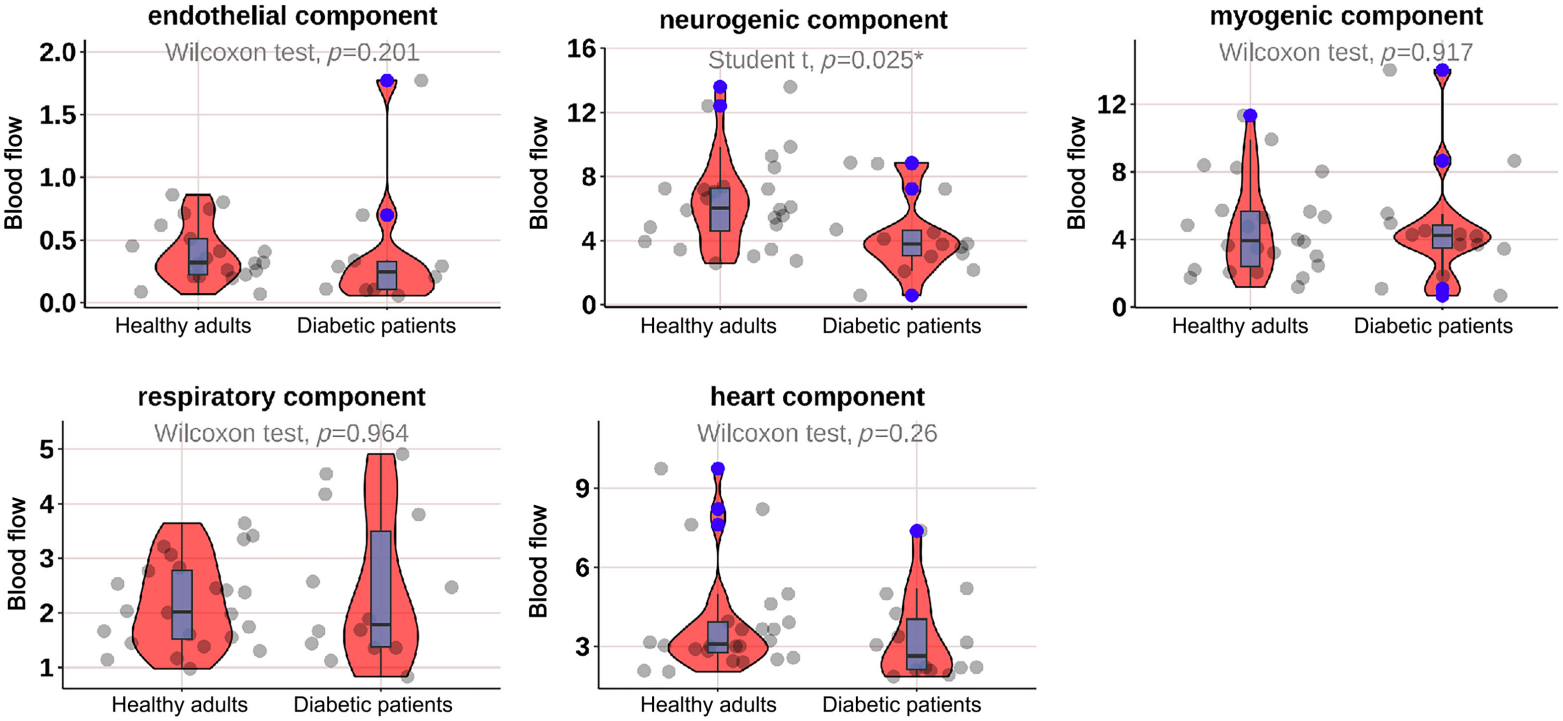
Violin plot comparing the time-integrated BF values of different wavelet components in the M5 region between diabetes patients and healthy adults. The neurogenic (p=0.025*) components in diabetes patients were significantly lower than those in healthy adults, whereas there was no significant difference in the endothelial, myogenic, respiratory, and heart components. The box plot represents the median and interquartile range, the gray scatter represents the BF of each subject, the blue scatter represents outliers, and the red kernel density plot represents the distribution of data density.

**Fig. 4:**
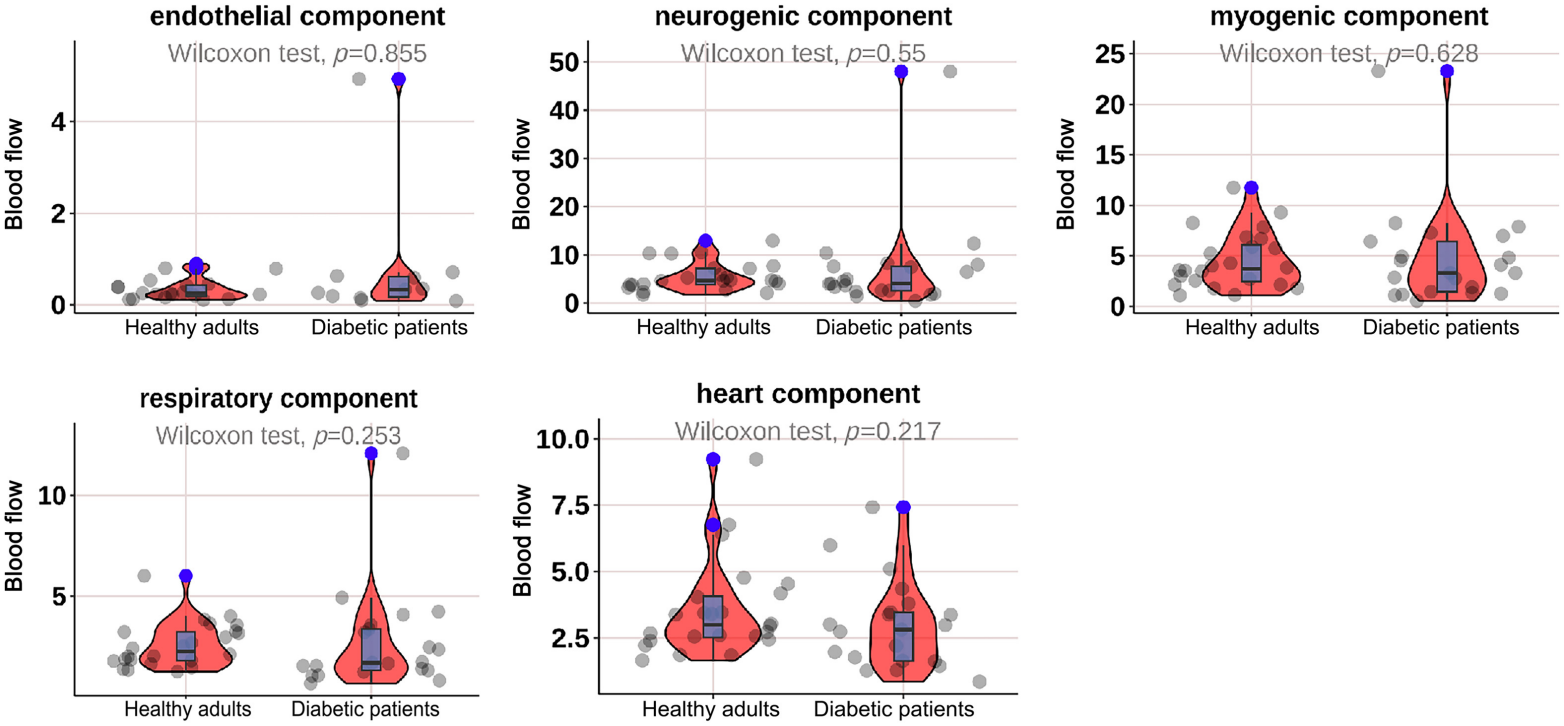
Violin plot comparing the time-integrated BF values of different wavelet components in the heel region between diabetes patients and healthy adults. There was no significant difference in the endothelial, neurogenic, myogenic, respiratory, and heart components. The box plot represents the median and interquartile range, the gray scatter represents the BF of each subject, the blue scatter represents outliers, and the red kernel density plot represents the distribution of data density.

**Fig. 5:**
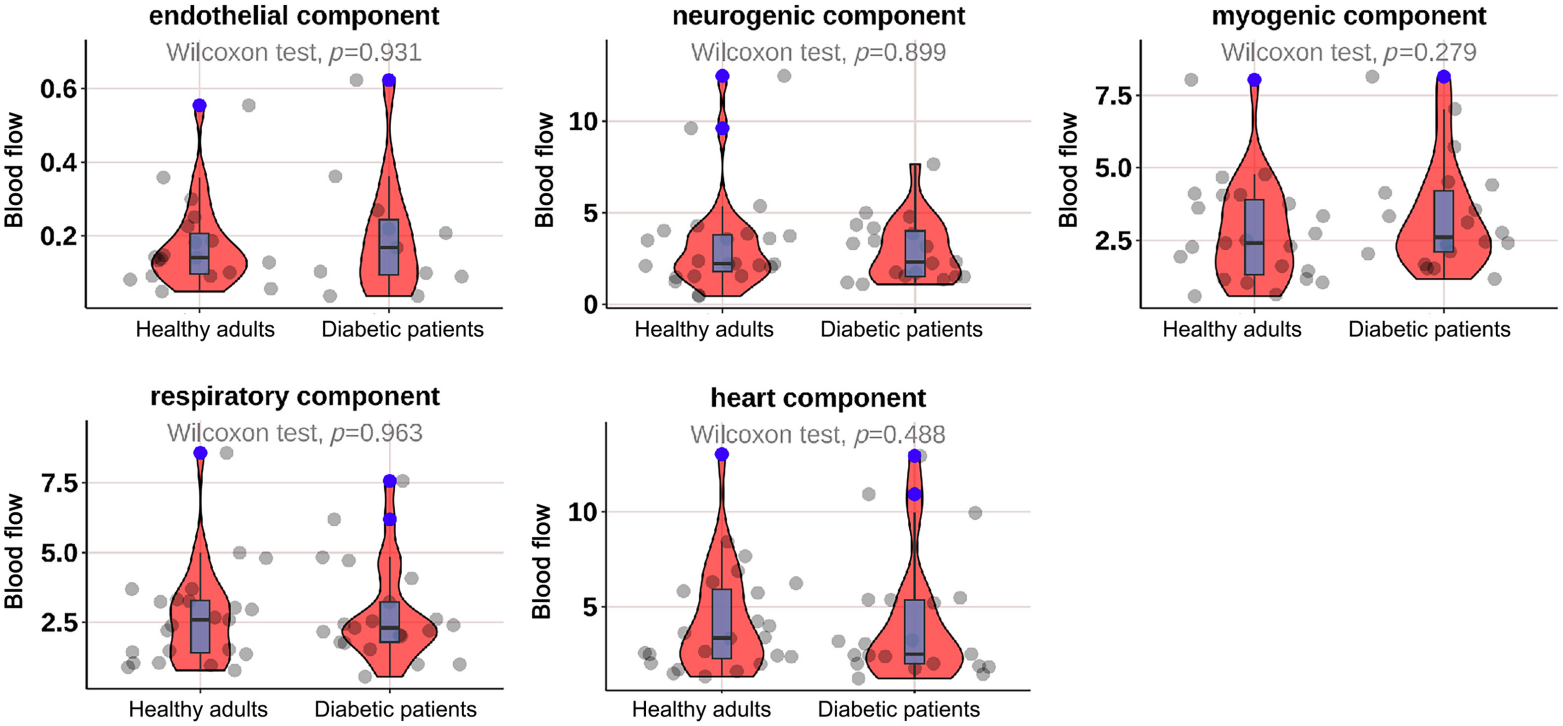
Violin plot comparing the time-integrated BF values of different wavelet components in the dorsum of the foot region between diabetes patients and healthy adults. There was no significant difference in the endothelial, neurogenic, myogenic, respiratory, and heart components. The box plot represents the median and interquartile range, the gray scatter represents the BF of each subject, the blue scatter represents outliers, and the red kernel density plot represents the distribution of data density.

As shown in Fig. 2, the mean BF in the neurogenic (p=0.044) and heart (p=0.001) components at M1 was significantly lower in diabetic patients; as shown in Fig. 3, the mean BF in the neurogenic component at the M5 was significantly lower in diabetic patients (p=0.025); as shown in Figs. 4 and 5, there was no significant difference in mean BF at the heel and dorsum of the foot between diabetic patients and healthy adults.

#### 3.1.1 Comparison of mean BF at four parts in healthy adults

**Fig. 6:**
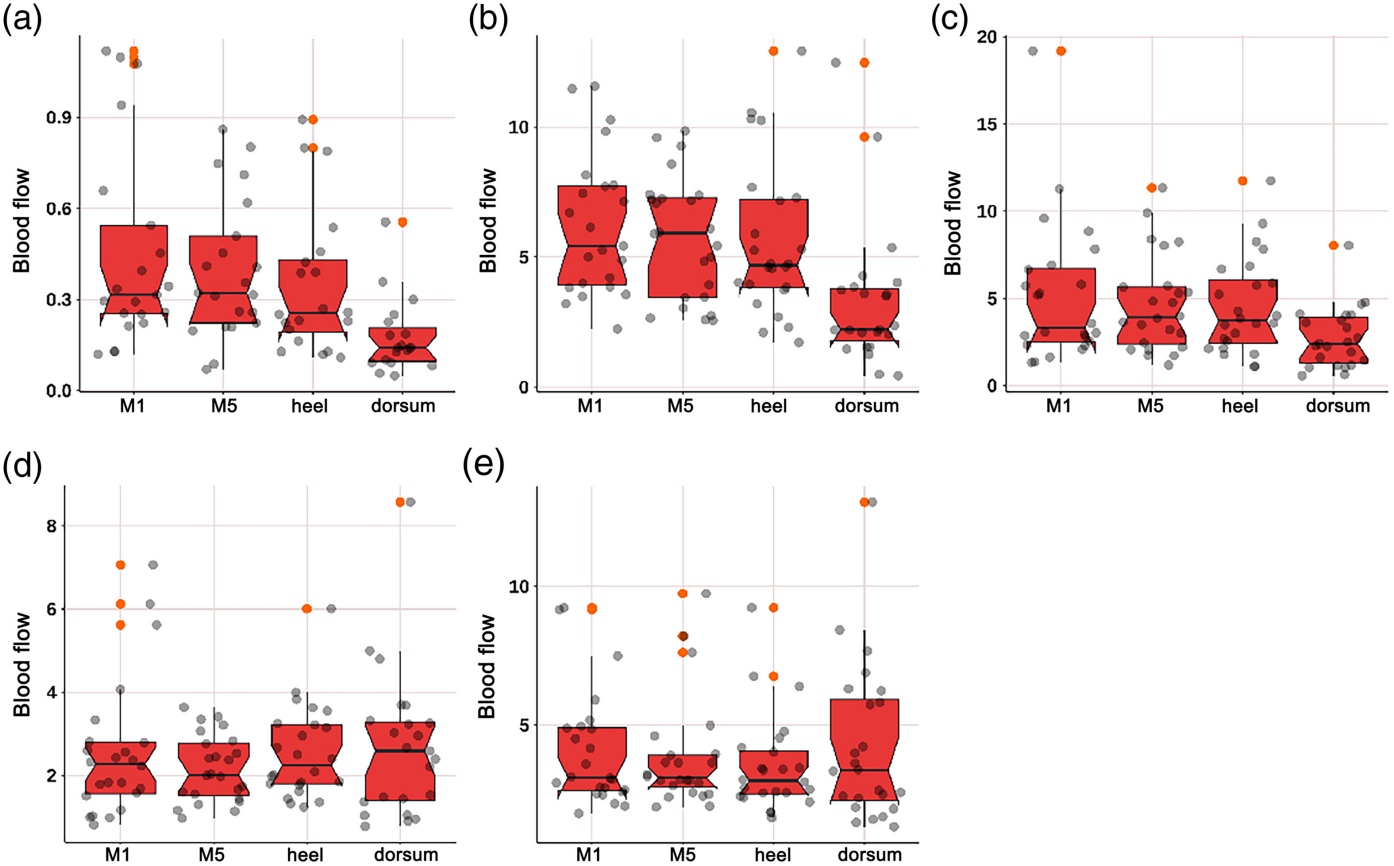
Box plot comparing the time-integrated BF values of different wavelet components among four regions of healthy adults. The endothelial, neurogenic, and myogenic components at the dorsum of the foot were lower than those at the three parts of the plantar, but the results were not statistically significant; there was no difference among the four parts in the respiratory and heart components.

As shown in Fig. 6, the endothelial, neurogenic, and myogenic components at the dorsum of the foot were lower than those at the three parts of the plantar, but the results were not statistically significant; there was no difference between the four parts in the respiratory and heart components.

#### 3.1.2 Comparison of mean BF at four parts in diabetic patients

**Fig. 7:**
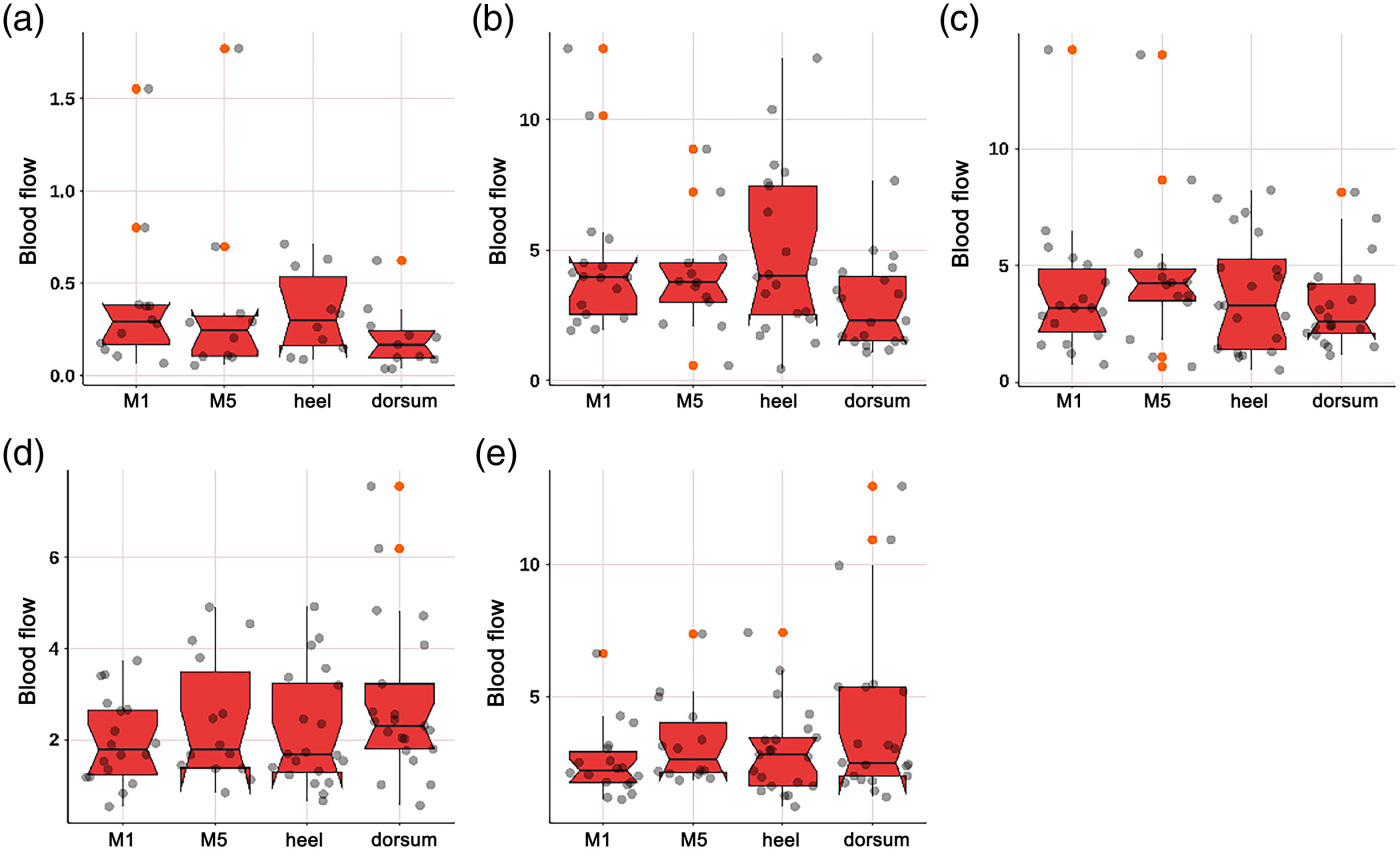
Box plot comparing the time-integrated BF values of different wavelet components among four regions of diabetic patients: (a) endothelial, (b) neurogenic, (c) myogenic, (d) respiratory, and (e) heart components. The neurogenic component at the dorsum of the foot was lower than that at the M1 area (p=0.056) and heel area (p=0.067), with marginal statistical significance. There were no significant differences in the endothelial, myogenic, respiratory, and heart components among the four regions.

As shown in Fig. 7, the neurogenic component at the dorsum of the foot was lower than that at the M1 area (p=0.056) and heel area (p=0.067), with marginal statistical significance.

### 3.2 Comparison of SE in Different Components Between Diabetic Patients and Healthy Adults

**Fig. 8:**
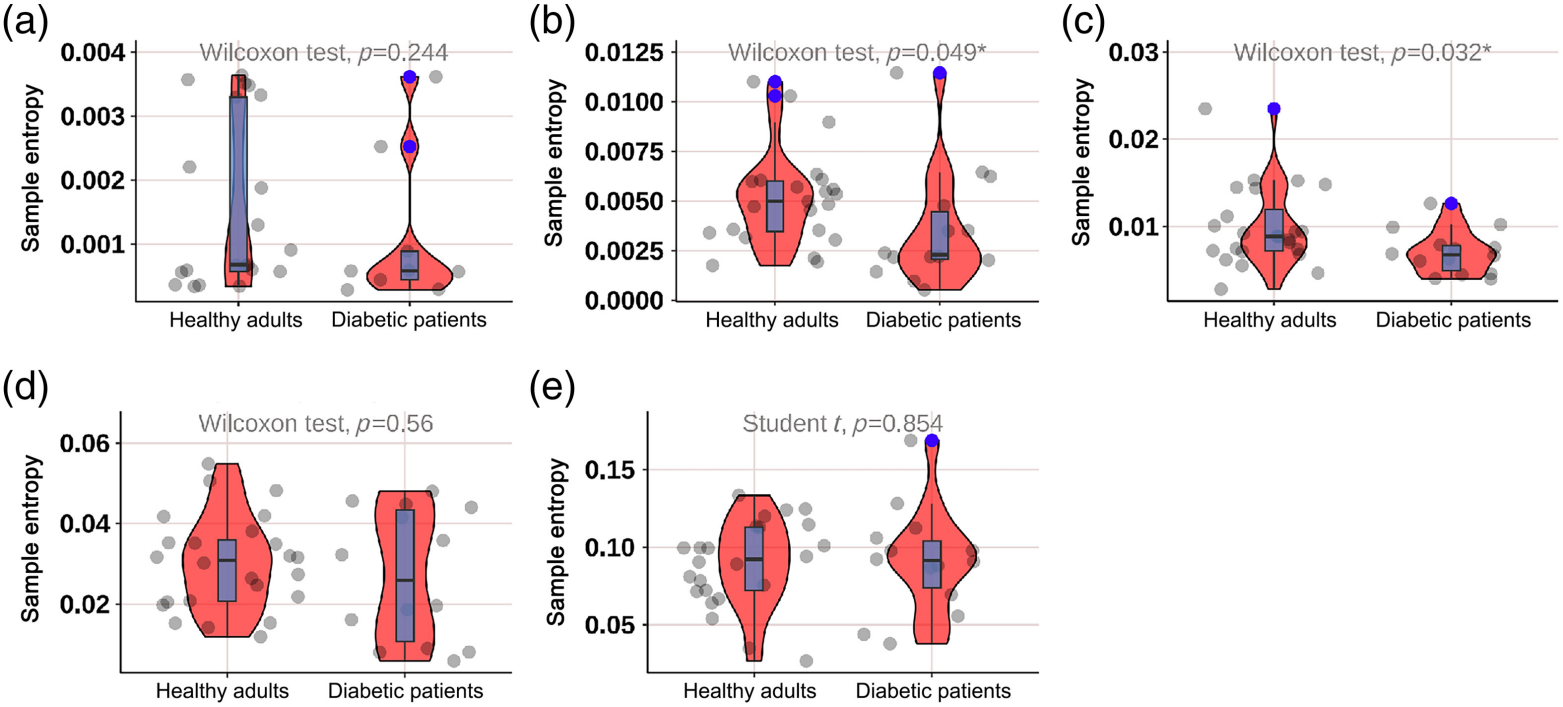
Violin plot comparing the time-integrated SE values of different wavelet components in the M1 region between diabetes patients and healthy adults: (a) endothelial, (b) neurogenic, (c) myogenic, (d) respiratory, and (e) heart components. The neurogenic (p=0.049) and myogenic (p=0.032) components in diabetes patients were significantly lower than those in healthy adults. There was no significant difference in the endothelial, respiratory, and heart components. The box plot represents the median and interquartile range, the gray scatter represents the BF of each subject, the blue scatter represents outliers, and the red kernel density plot represents the distribution of data density.

**Fig. 9:**
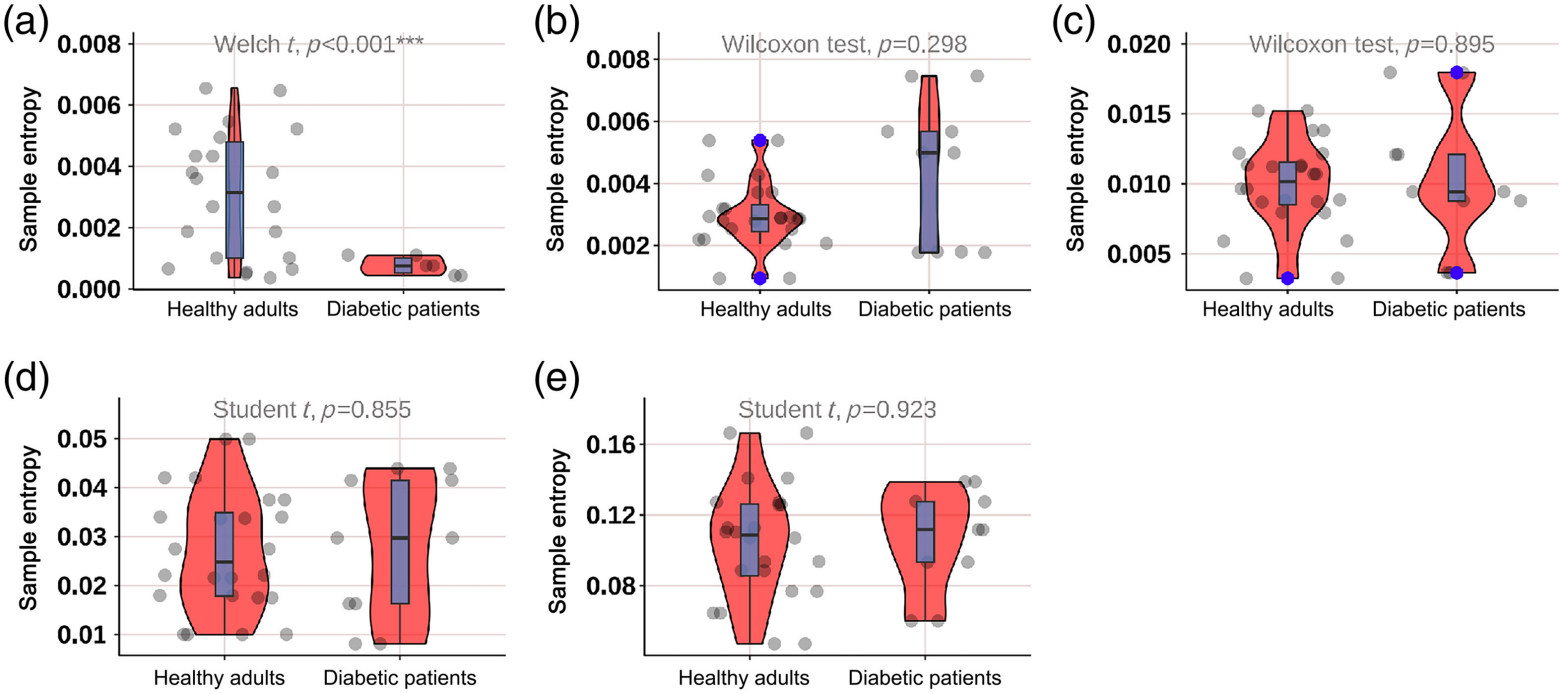
Violin plot comparing the time-integrated SE values of different wavelet components in the M5 region between diabetes patients and healthy adults: (a) endothelial, (b) neurogenic, (c) myogenic, (d) respiratory, and (e) heart components. The endothelial (p<0.001) components in diabetes patients were significantly lower than those in healthy adults. There was no significant difference in the neurogenic, myogenic, respiratory, and heart components. The box plot represents the median and interquartile range, the gray scatter represents the BF of each subject, the blue scatter represents outliers, and the red kernel density plot represents the distribution of data density.

**Fig. 10:**
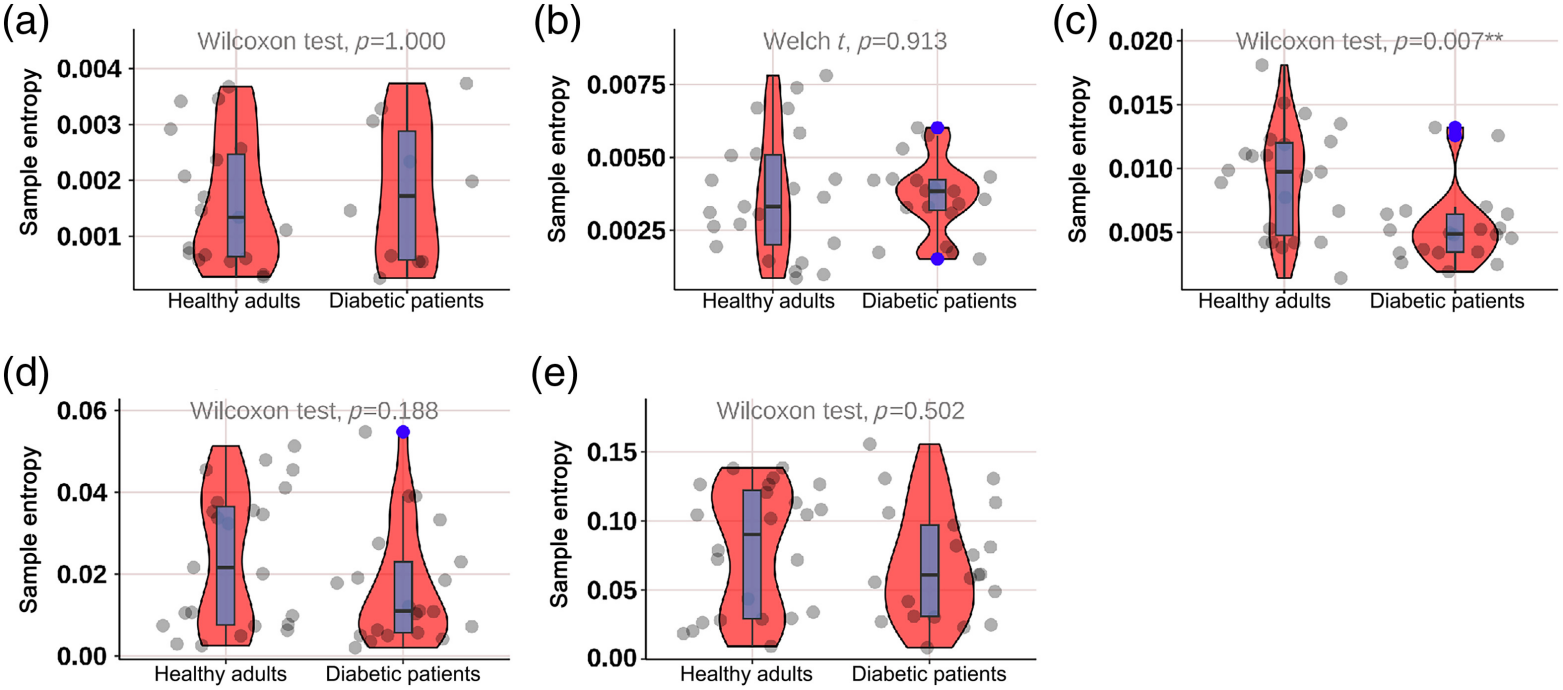
Violin plot comparing the time-integrated SE values of different wavelet components in the dorsum of the foot region between diabetes patients and healthy adults: (a) endothelial, (b) neurogenic, (c) myogenic, (d) respiratory, and (e) heart components. The myogenic (p=0.007) component in diabetes patients was significantly lower than those in healthy adults. There was no significant difference in the endothelial, neurogenic, respiratory, and heart components. The box plot represents the median and interquartile range, the gray scatter represents the BF of each subject, the blue scatter represents outliers, and the red kernel density plot represents the distribution of data density.

**Fig. 11:**
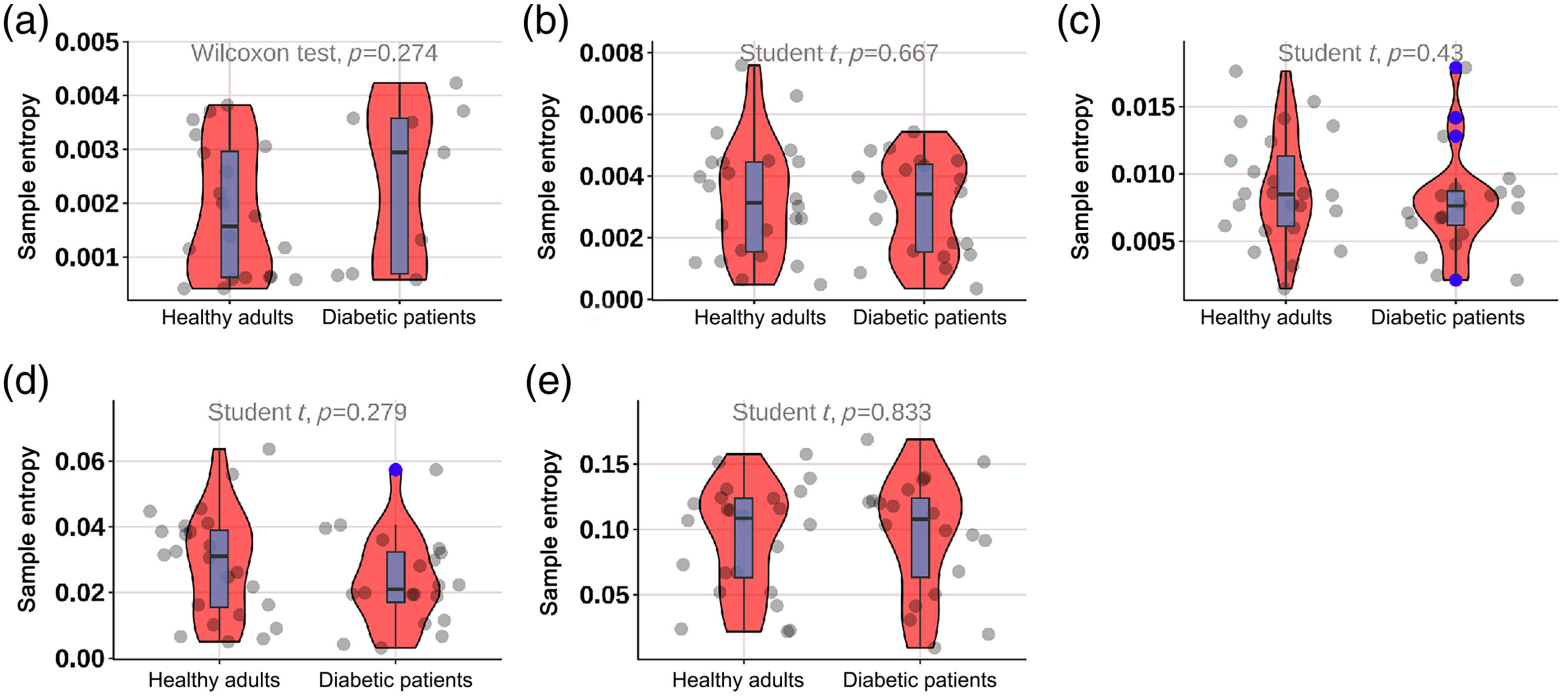
Violin plot comparing the time-integrated SE values of different wavelet components in the heel region between diabetes patients and healthy adults: (a) endothelial, (b) neurogenic, (c) myogenic, (d) respiratory, and (e) heart components. There was no significant difference in the endothelial, neurogenic, myogenic, respiratory, and heart components. The box plot represents the median and interquartile range, the gray scatter represents the BF of each subject, the blue scatter represents outliers, and the red kernel density plot represents the distribution of data density.

As shown in Fig. 8, the SE of diabetic patients in the neurogenic (p=0.049) and myogenic (p=0.032) components at M1 was significantly lower; as shown in Fig. 9, the SE of diabetic patients in the endothelial (p<0.001) component at M5 was significantly lower; as shown in Fig. 10, the SE of diabetic patients in the myogenic component at the dorsum of the foot was significantly lower (p=0.007); as shown in Fig. 11, there was no difference in SE at the heel between diabetic patients and healthy adults.

#### 3.2.1 Comparison of SE at four parts in healthy adults

**Fig. 12:**
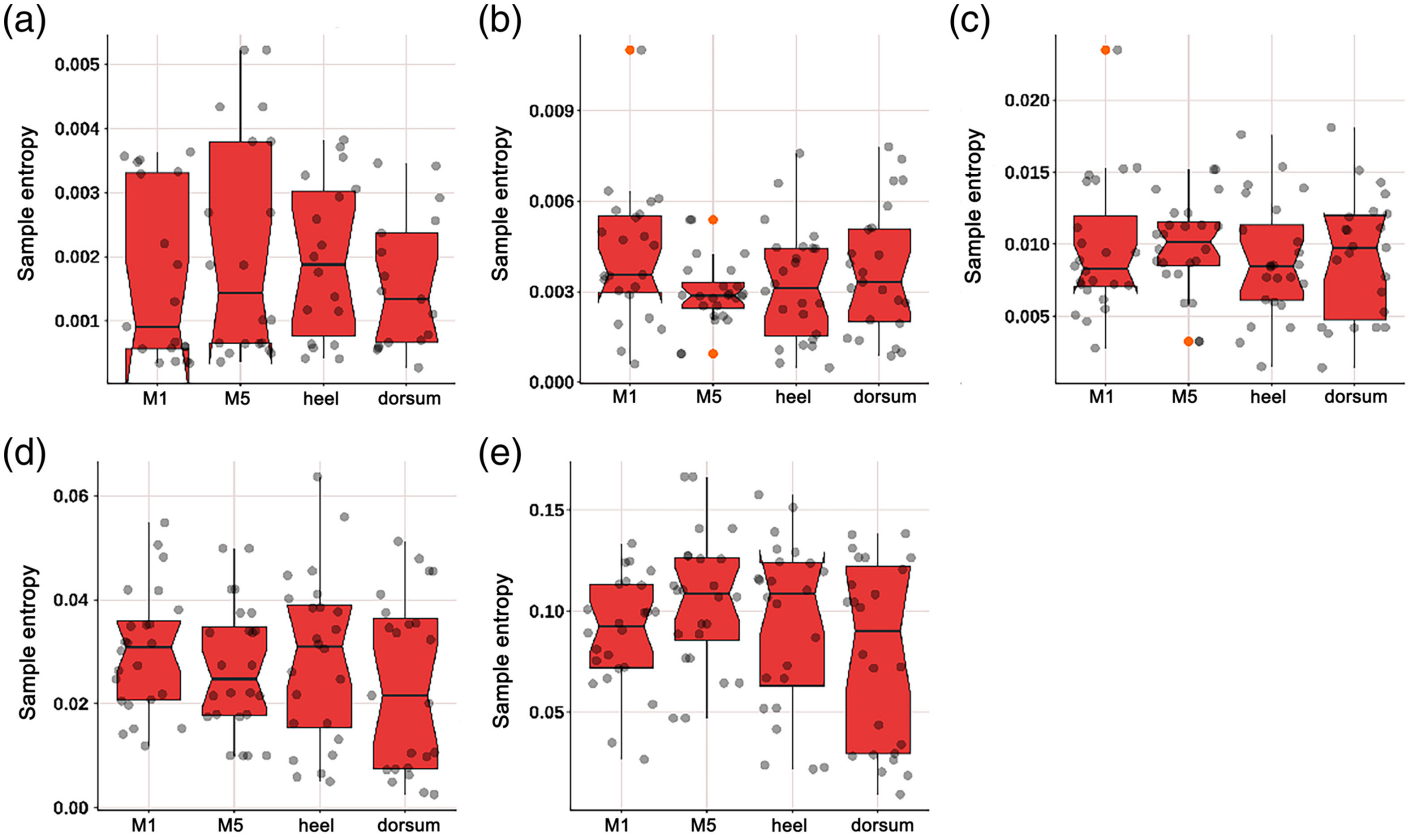
Box plot comparing the time-integrated SE values of different wavelet components among four regions of healthy adults: (a) endothelial, (b) neurogenic, (c) myogenic, (d) respiratory, and (e) heart components. There were no significant differences in the endothelial, neurogenic, myogenic, respiratory, and heart components among the four regions.

As shown in Fig. 12, there was no difference in SE among different parts.

#### 3.2.2 Comparison of SE at four parts in diabetic patients

**Fig. 13:**
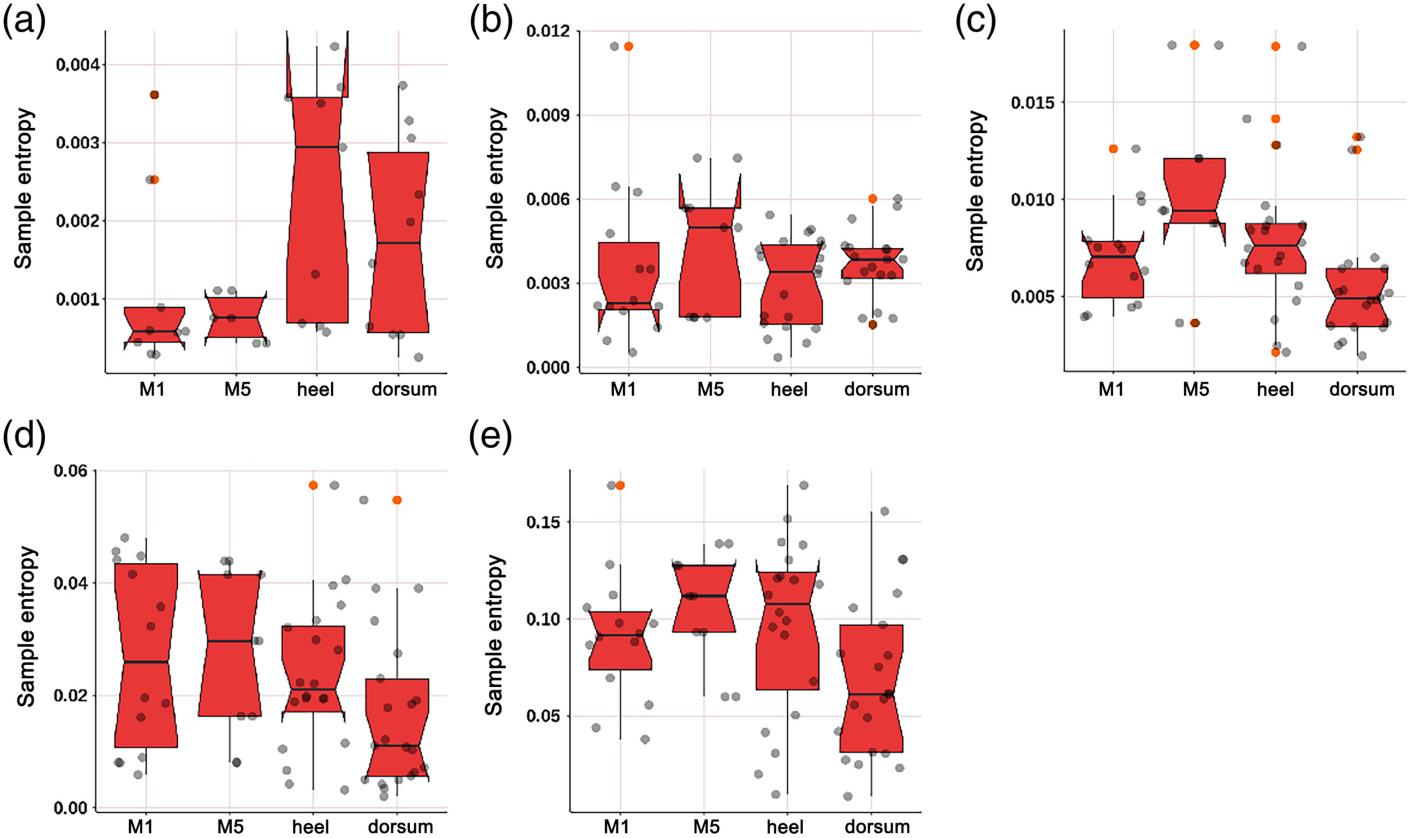
Box plot comparing the time-integrated SE values of different wavelet components among four regions of diabetes patients: (a) endothelial, (b) neurogenic, (c) myogenic, (d) respiratory, and (e) heart components. The myogenic component at the dorsum of the foot was lower than that at the M5 area (p=0.050) and heel area (p=0.041). The heart component at the dorsum of the foot was significantly lower than that at the M5 area (p=0.017) and heel area (p=0.028). There were no significant differences in the endothelial, neurogenic, and respiratory components among the four regions.

As shown in Fig. 13, the myogenic component at the dorsum of the foot was lower than that at the M5 area (p=0.050) and heel area (p=0.041); the heart component at the dorsum of the foot was lower than that at the M5 area (p=0.017) and heel area (p=0.028).

## 4 Discussion

Our study demonstrated that the foot microcirculation of diabetic patients was significantly lower than that of healthy adults, mainly in the plantar areas near the distal end of the foot (first and fifth metatarsal bones), with the wavelet components mainly in the heart and neurogenic origins; the SE of BF in diabetic patients was significantly lower than that in healthy adults in all areas except the heel, involving the myogenic, neurogenic, and endothelial components, indicating that the BF changes during BF monitoring in diabetic patients were small, which may be related to their impaired self-regulation mechanism; among the four test areas, the average BF at the dorsum of the foot was significantly lower than that at the plantar, and the SE at the dorsum of the foot was lower in diabetic patients. To our knowledge, this is the first study to use wearable LDF and complexity analysis to compare the local difference of plantar BF oscillations between diabetic patients and healthy adults. Wearable LDF is not limited by the test site and can complete the test conveniently and quickly. Our study lays a preliminary foundation for the future development of wearable plantar microcirculation detection.

The areas where diabetic patients are prone to develop ulcers are: lateral side of M1, under the first to fifth metatarsal heads, medial side of midfoot, and heel.^21^ Our study found that compared with healthy adults, the mean BF in different components of diabetic patients was significantly lower in the neurogenic and heart components at M1 and in the neurogenic component at M5, which was consistent with the areas where diabetic patients were prone to develop foot ulcers. The neurogenic component was lower at both M1 and M5. According to the definition of wavelet analysis, this result reflects that the neurogenic regulation mechanism of blood vessels is impaired. The ABI (healthy adults 1.16±0.11, diabetic patients 1.17±0.08) of both groups of patients was within normal range, indicating no ischemia due to lower limb arterial disease. It can be inferred that the factor causing the decrease of mean BF at M1 and M5 is the impaired neurogenic regulation mechanism of blood vessels. In the study of Jan et al.^10^ involving 18 DM2 with peripheral neuropathy and 8 healthy controls, LDF was used to measure skin BF at M1 under 300 mmHg mechanical stress and 42°C rapid thermal stress. Wavelet analysis of skin BF oscillations was used to evaluate metabolic, neurogenic, and myogenic control. The study applied pressure and thermal stimuli under M1 but could not simulate the stress under real walking conditions. The results also found that diabetic patients had significantly reduced metabolic, neurogenic, and myogenic responses to thermal stress, which was consistent with our study results, but the authors did not analyze the difference in SE in wavelet components or compare between groups. Mizeva et al.^12^ studied 40 healthy subjects, 17 DM1 and 23 DM2. Skin BF was collected by LDF. One foot was cooled to 25°C for 4 min, and local thermal tests were performed at 35°C and 42°C for 4 and 10 min, respectively. The results suggested that local temperature tests showed impaired vascular dilation function in response to local heating in diabetic patients. A trend of impaired low-frequency BF pulsations related to endothelial and neurogenic activity was observed in both groups of diabetic patients. The results were consistent with our findings, but it was difficult to compare between two groups of patients because DM1 were mostly young people, whereas DM2 were usually middle-aged and elderly people. The inevitable difference in age may affect the study results. Combining previous studies, we speculate that before peripheral neuropathy can be clearly diagnosed by conventional examination methods, there is already a neuropathy that regulates microcirculation in diabetic patients with early symptoms of peripheral neuropathy. This inference needs to be confirmed by future large-sample studies, but it can be clearly stated that LDF can detect microcirculation damage earlier than conventional examination methods.

Our study found that the neurogenic component at the dorsum of the foot was lower than that at the M1 area (p=0.056) and heel area (p=0.067) in diabetic patients, with marginal statistical significance. The SBF of the plantar in Diabetic Peripheral Neuropathy (DPN) patients was higher, which seems to indicate that diabetic patients have a better BF supply to the plantar, which may mean a lower risk of developing DFUs. The specific reasons are analyzed as follows: The BF regulation mechanisms of the dorsum and plantar of the human foot are different. Hairless skin (e.g., plantar) has a rich network of arteriovenous anastomoses (AVAs), which are innervated by sympathetic adrenergic vasoconstrictor nerves.^22^ AVAs can divert blood from small arteries to small veins without capillary connections, thereby reducing BF to capillaries. Hairy skin (e.g., dorsum of the foot) has two types of sympathetic nerve-mediated reflex control, including a noradrenergic vasoconstrictor system and a cholinergic (active) sympathetic vasodilator system. Under the removal of sympathetic nerve control, Wilson et al.^23^ showed that hairless skin can regulate BF automatically during blood pressure changes, whereas hairy skin cannot show BF autoregulation. In this study, DPN patients were included, who may lose the sympathetic adrenergic vasoconstrictor nerve function that regulates the plantar skin AVAs.^18^ Studies have confirmed that 80% to 90% of the BF in the plantar circulates through AVAs, and 10% to 20% circulates through the nutritional capillary bed.^24^ Therefore, theoretically, the plantar BF of diabetic patients with peripheral neuropathy is higher than that of the dorsum of the foot (without AVAs) and also higher than that of healthy non-diabetic controls (AVAs are still under active sympathetic adrenergic vasoconstrictor nerve control).^25^ This is because the loss of vasoconstriction of small arteries leads to reduced vascular resistance and increased BF through AVAs.^26^

Our study found that compared with healthy adults, the SE of diabetic patients in different components was significantly lower; as shown in Fig. 7, the SE of diabetic patients in the neurogenic (p=0.049) and myogenic (p=0.032) components at M1 was significantly lower; as shown in Fig. 8, the SE of diabetic patients in the endothelial (p<0.001) component at the M5 was significantly lower; as shown in Fig. 9, the SE of diabetic patients in the myogenic component at the dorsum of the foot was significantly lower (p=0.007); overall, the SE of BF in diabetic patients was significantly lower than that in healthy adults in all areas except the heel, which suggests that the vascular smooth muscle, nerve, and endothelial regulation mechanisms are impaired, which is consistent with the decrease of mean BF at the first and fifth metatarsal bones and dorsum of the foot in diabetic patients. Certainly, it is important to emphasize that despite the utilization of EEMD in preprocessing, which effectively detected significant disparities between DM patients and healthy controls, a 3-min recording duration remains relatively brief for comprehensive analysis of low-frequency components. Therefore, drawing upon the valuable comparative insights from our study on different measurement locations, we strongly advocate extending the recording time in future research endeavors to enhance the robustness and reliability of findings related to such components. The outcome indicators of our study revealed that the diabetic patients included exhibited lower levels compared to healthy adults, indicating a high risk for ulceration in the region. Additionally, there was a significant reduction observed in the neurogenic component of both outcome measures. Due to the design of our study, we could not further evaluate the severity of neurogenic impairment. Future studies may need to use LDF to quantify the severity of neurogenic impairment in different components, rather than using 10-g nylon filaments to diagnose peripheral neuropathy. Other methods can also be used, including postural changes to examine the regulation of various BF controls at the plantar and dorsum of the foot, such as standing and natural walking. Our study also found that compared with four parts in diabetic patients, as shown in Fig. 11, the myogenic component at the dorsum of the foot was lower than that at the M5 area (p=0.050) and heel area (p=0.041); the heart component at the dorsum of the foot was lower than that at the M5 area (p=0.017) and heel area (p=0.028), indicating that the SE of the dorsum of the foot in diabetic patients was lower than that of the plantar and that the muscle and heart regulation mechanisms at the dorsum of the foot were more regular. When diabetic patients’ feet were subjected to pressure or thermal stimuli, the muscle and heart regulation mechanisms at the dorsum of the foot could not respond accordingly, which also illustrates again the difference between BF regulation mechanisms at the plantar and dorsum of the foot. Park et al.^27^ demonstrated that diabetic patients lost their normal constriction response when standing. This may further explain the difference in the degree of neuropathy involvement between plantar (sympathetic adrenergic vasoconstrictor nerve) and dorsum (noradrenergic vasoconstrictor nerve and cholinergic sympathetic vasodilator system) skin in DPN patients. In the study of Jan et al.^10^ involving 18 type 2 diabetic DPN patients and 8 healthy controls, SE analysis was used to quantify the regularity degree of skin BF oscillations. SE analysis showed that diabetic DPN patients had a higher regularity of plantar skin BF than dorsum foot, which contradicted our study results. The possible reason for this discrepancy is that this study did not analyze SE in different components in detail, so there were differences in results. Our analysis of SE in different components helps to qualitatively analyze microcirculation damage regulation mechanisms.

Our study also has limitations. We compared mean BF and SE at different parts under resting state between diabetic patients and healthy adults. Although microvascular reactivity is considered to better characterize BF control mechanism damage in diabetic patients,^23^ these large vessel reactivity tests (e.g., thermal congestion and reactive congestion) take a long time to complete, which may limit their role in screening diabetic patients with risk of foot ischemia and ulceration. Future studies need plantar wearable walking LDF devices to compare different microvascular reactivity; second, our sample size was small, which may affect the reliability of our study results. Future studies need to include large samples of diabetic patients.

## 5 Conclusions

Wearable LDF has a unique advantage in rapidly assessing plantar microcirculation in the early stage of diabetic ulceration. Combined with wavelet analysis, it can quantitatively and qualitatively analyze the regulation mechanism of foot microcirculation. Compared with healthy adults, diabetic patients had significantly lower foot microcirculation, mainly in the plantar areas near the distal end of the foot, with the wavelet components mainly in the heart and neurogenic origins; diabetic patients had significantly lower BF SE in all areas except the heel, involving the myogenic, neurogenic, and endothelial components, indicating that it was related to the impairment of BF regulation mechanism of diabetes itself. Early intervention in the muscle, nerve, and endothelial function of diabetic patients’ feet may be an effective way to improve foot microcirculation and prevent DFUs.

## Supplementary Material



## Data Availability

Code and data will be made available upon request.
