# Supplementary material for: Wearable laser Doppler flowmetry for non-invasive assessment of diabetic foot microcirculation: methodological considerations and clinical implications
Source: J Biomed Opt. 2024 May 11;29(6):065001. doi: 10.1117/1.JBO.29.6.065001 (PMC11088439; doi:10.1117/1.JBO.29.6.065001)
Supplement: Supplementary file 1 [file JBO_029_065001_SD001.pdf]

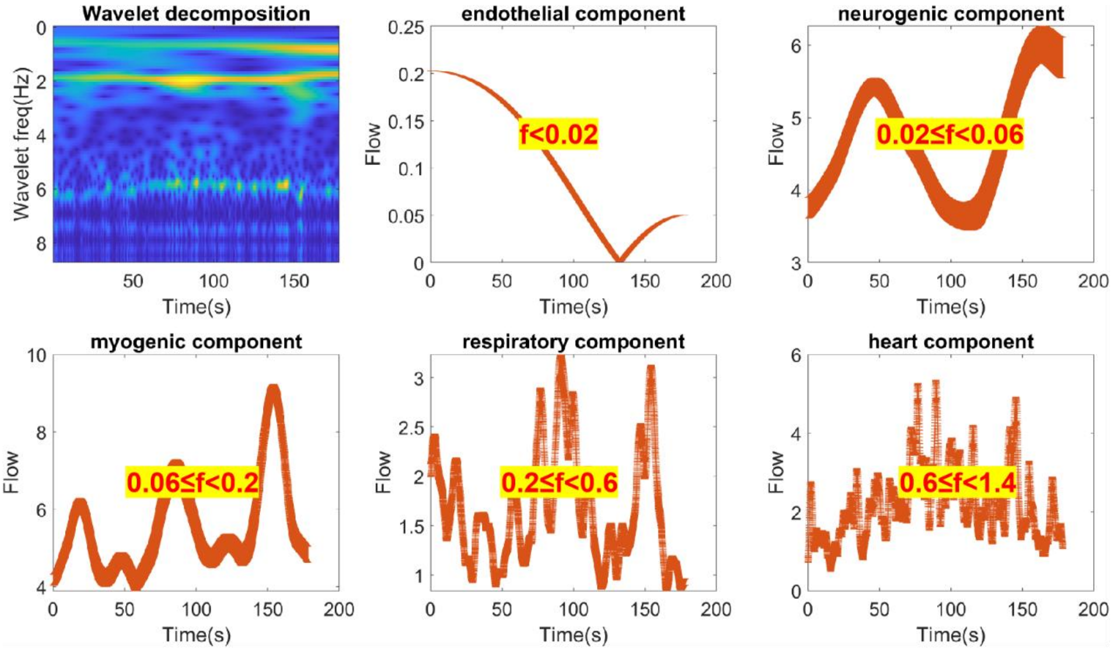

Figure S1: Typical wavelet decomposition of one subject in this study, and wavelet decomposition of five individual control mechanisms: endothelial origin (＜0.02 Hz), neurogenic origin (0.02-0.06 Hz), myogenic origin (0.06-0.2 Hz), respiratory origin (0.2-0.6 Hz), and heart origin (0.6-1.4 Hz).

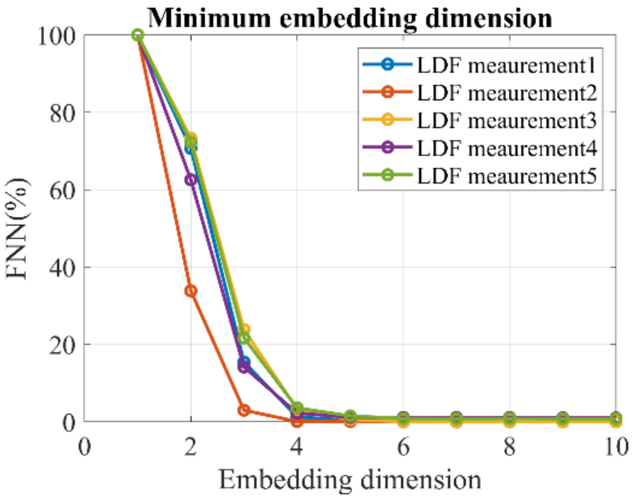

Figure S2: Estimation of the minimum embedding dimension of LDF by the false nearest neighbor (FNN) method.

Table S1. Test procedures conducted to assess various sensory functions of the foot

Tests	Detailed procedures
Skin Touch Test (10g-Semmes-Weinstein Nylon Monofilament)	Ten parts of the plantar and dorsum of the foot were examined;<br>Each part was scored 1 point if it was normal;<br>The total score ranged from 0 to 10 points.<br>A full score ≤8 points indicated abnormal skin touch of the foot.
Skin Vibration Sense (Ryder-Seiffer Semi-Quantitative Vibration Tuning Fork)	Three areas were tested: the dorsal side of the distal phalanx of the thumb, the dorsal side of the fifth metatarsal head, and the tip of the medial malleolus.<br>If the tuning fork reading was <5 in any of these areas, it indicated abnormal vibration sense of the foot.
Temperature Sense (Temperature Sensation Tester)	The test site was the same as the monofilament touch test.<br>If the total score ≤8 points, it indicated abnormal temperature sense of the foot.
